# EDUCATION, SOCIAL NETWORKS, AND COGNITION IN CROSS-NATIONAL CONTEXT

**DOI:** 10.1093/geroni/igae098.1536

**Published:** 2024-12-31

**Authors:** James Iveniuk, Markus Schafer, Haosen Sun

**Affiliations:** The National Opinion Research Center, Chicago, Illinois, United States; Baylor University, Waco, Texas, United States; University of Nevada, Reno, Reno, Nevada, United States

## Abstract

Previous research has shown that older adults with more education report both larger social networks and better cognitive function. But educational institutions vary greatly from country to country, meaning the process whereby education conserves cognition through social networks may vary by national context. In this paper, we bring together data from the National Social Life Health and Aging Project (NSHAP, 4777 cases collected in 2015/2016) and the Survey of Health Ageing and Retirement in Europe (SHARE; 67200 cases across 18 countries collected in 2015) to examine associations between education and cognition cross-nationally, and how much of the association between education and cognition is mediated by social network characteristics (i.e. size and proportion kin). We standardize education using the International Standard Classification of Education 1997. We find that across every country, more education is associated with better cognition. In most countries, education is also positively associated with network size, and negatively associated with proportion kin. Network size is generally positively associated with cognition, and proportion kin is negatively associated with cognition. We find significant indirect effects for network size of in all but four countries (Czechia, Spain, Greece, Israel), with the United States showing one of the largest indirect effect sizes. Overall, the proportion mediated by network size was small across all countries – 7.4% at maximum, and 4.1% across all countries. We also found fewer and weaker indirect effects with proportion kin. We conclude with suggestions for harmonization analysis and implications for networks, international educational stratification, and cognitive aging.

